# Molecular Cloning of *HbPR-1* Gene from Rubber Tree, Expression of *HbPR-1* Gene in *Nicotiana benthamiana* and Its Inhibition of *Phytophthora palmivora*

**DOI:** 10.1371/journal.pone.0157591

**Published:** 2016-06-23

**Authors:** Uraiwan Khunjan, Kitiya Ekchaweng, Tanate Panrat, Miaoying Tian, Nunta Churngchow

**Affiliations:** 1 Department of Biochemistry, Faculty of Science, Prince of Songkla University, Hat Yai, Songkhla, Thailand; 2 Department of Plant and Environmental Protection Sciences, University of Hawaii at Manoa, Manoa, HI, United States of America; 3 Digital Media Program, Prince of Songkla University International College, Prince of Songkla University, Hat Yai, Songkhla, Thailand; 4 Center for Genomics and Bioinformatics Research, Faculty of Science, Prince of Songkla University, Hat Yai, Songkhla, Thailand; USDA/ARS, UNITED STATES

## Abstract

This is the first report to present a full-length cDNA (designated *HbPR-1*) encoding a putative basic HbPR-1 protein from rubber tree (*Hevea brasiliensis*) treated with salicylic acid. It was characterized and also expressed in *Nicotiana benthamiana* using *Agrobacterium*-mediated transient gene expression system in order to investigate the role of *HbPR-1* gene in rubber tree against its oomycete pathogen *Phytopthora palmivora* and to produce recombinant HbPR-1 protein for microbial inhibition test. The *HbPR-1* cDNA was 647 bp long and contained an open reading frame of 492 nucleotides encoding 163 amino acid residues with a predicted molecular mass of 17,681 Da and an isoelectric point (pI) of 8.56, demonstrating that HbPR-1 protein belongs to the basic PR-1 type. The predicted 3D structure of HbPR-1 was composed of four α-helices, three β-sheets, seven strands, and one junction loop. Expression and purification of recombinant HbPR-1 protein were successful using *Agrobacterium*-mediated transient expression and one-step of affinity chromatography. Heterologous expression of *HbPR-1* in *N*. *benthamiana* reduced necrosis areas which were inoculated with *P*. *palmivora* zoospores, indicating that the expressed HbPR-1 protein played an important role in plant resistance to pathogens. The purified recombinant HbPR-1 protein was found to inhibit 64% of *P*. *palmivora* zoospore germination on a water agar plate compared with control, suggesting that it was an antimicrobial protein against *P*. *palmivora*.

## Introduction

Growth and productivity of plants depend on their ability in responding and adapting to external stresses. Plants defend themselves against external stresses such as pathogen invasion by changing gene expression which leads to the synthesis of specific proteins [1–6]. Pathogenesis-related (PR) proteins which are implicated in inhibiting growth, multiplication and/or spread of pathogens are induced and accumulate in host plants [7–9]. PR proteins have so far been classified into 17 different families [10]. Even though the biological activity of PR-1 protein remains elusive, it has been frequently used in many plant species as a marker for systemic acquired resistance (SAR) [2,6–7,11–15].

PR-1 proteins are synthesized by plants in response to pathogen infections, salicylic acid (SA), ethylene and environmental stresses [1,6–7,14–17]. Plant PR-1 proteins are classified into two groups as acidic and basic forms based on their isoelectric points (pI) [7]. PR-1 proteins share similar structure characteristics. The primary translation products of the *PR-1* genes contain a hydrophobic signal sequence. The mature PR-1 proteins contain six conserved cysteine residues forming disulfide bridges, four α-helices and one β-sheet formed by four β-strands [7,10].

Although there were many studies on PR-1 proteins in several plant species, a study of PR-1 in *H*. *brasiliensis* (rubber tree) has not been reported. Rubber tree, a perennial tropical plant, is the major source of natural rubber in the world [18] and an economically important crop of Thailand which is known as the world leading producer and exporter of natural rubber. This is the first report to present a full-length cDNA (designated *HbPR-1*) encoding a putative basic HbPR-1 protein from rubber tree. It was characterized and also expressed in *N*. *benthamiana* using *Agrobacterium*-mediated transient gene expression system in order to investigate the function related to plant defense and its antimicrobial activity. This system is a fast, flexible, scalable and reproducible method for analysis of gene functions [19–20]. As compared to the stable gene expression, transient gene expression system offers a number of advantages. One of the most important advantages is its simplicity and easy performance [19]. Transient expression in several different plant species has been described. Among these species, *N*. *benthamiana* is widely used as a model plant in many plant research laboratories. Moreover, proteins can be easily expressed at high levels in *N*. *benthamiana* through leaf agroinfiltration and the process is very simple and time-saving without the need of complicated equipment [19,21–24]. Since this system is limited by post-transcriptional gene silencing (PTGS), co-expression of a viral-encoded suppressor of gene silencing, such as p19 protein of tomato bushy stunt virus (TBSV), is used to suppress the onset of PTGS in the infiltrated tissues and to enhance expression levels of recombinant proteins in plants [22,25–26].

Due to no antibody available to directly monitor PR-1 proteins, isolation and purification of these proteins using classical biochemical approaches are difficult. Furthermore, direct purification of endogenous PR-1 proteins from the rubber tree plants is time-consuming since they have long growth cycle and low protein expression levels. Therefore, a recombinant DNA technology is likely to be a suitable choice for producing a large amount of PR-1 proteins for further application in agriculture, industry and pharmacy.

The objectives of our study are isolation, molecular cloning and characterization of *HbPR-1* from rubber tree seedlings treated with salicylic acid. Transient gene co-expression with p19 via *Agrobacterium* co-infiltration into *N*. *benthamiana* leaves was carried out to investigate the role of *HbPR-1* gene in rubber plant against *P*. *palmivora* and to produce recombinant HbPR-1 protein for microbial inhibition test.

## Materials and Methods

### Plant material

Bud-grafted rubber seedlings (*H*. *brasiliensis* cv. RRIM600) were grown in a field for 21 days and transferred to a controlled room in which the plants were exposed to a photoperiod of 12 h light and 12 h dark for 1 day. The 22-day-old rubber seedlings were sprayed with 4 mM salicylic acid (SA) solution to induce production of PR-1 proteins. Rubber leaves were collected 24 h after SA treatment for total RNA extraction.

*N*. *benthamiana* seedlings were grown in pots under growth chamber conditions at 25°C, 60% humidity, 16 h light/8 h dark cycle for 6 weeks.

### Preparation of *Phytophthora palmivora* zoospores

*P*. *palmivora* PP3-3 isolated from a diseased papaya plant was cultured on 10% unclarified V8 agar medium at room temperature for 5 days. The culture was covered with sterile distilled water and incubated at 4°C for 30 min then shaken at 50 rpm at room temperature for 30 min to release the motile zoospores.

### Amplification and cloning of *HbPR-1*

Molecular cloning of *HbPR-1* consisted of three steps: Amplification of *HbPR-1* partial fragment by PCR, 3′ and 5′ rapid amplification of cDNA ends (RACE) PCR and *HbPR-1* full-length cDNA amplification. Total RNA was extracted from leaves of rubber plants which were treated with 4 mM SA for 24 h using RNeasy^®^Plant Mini Kit (Qiagen, https://www.qiagen.com) following the manufacturer’s instructions. The total RNAs were treated with RNase-free DNaseI (Qiagen) at 37°C for 30 min to eliminate genomic DNA. The reaction was inactivated by heating at 65°C for 10 min. The purity of total RNAs was confirmed based on A260 nm/A280 nm ratio >1.8. The first-stranded cDNAs were synthesized using the SuperScript III reverse transcriptase RT-PCR system (Invitrogen, https://www.thermofisher.com) according to the manufacturer’s instructions. PCR was performed with the degenerate primers, dFPR-1: 5′-TTCACAACCAGGCACGAGS(G/C)A GCGGT-3′ and dRPR-1: 5′-ATGGTTCCACCATTGTTACACCTCAC-3′ using *Taq* DNA polymerase (Invitrogen). The degenerate primers were designed based on the conserved sequences of two *PR-1* genes (*Arabidopsis thaliana*: NM_127025.2 and *Eutrema wasabi*: AB271488.1). The reaction was performed with PCR condition of initial denaturation step at 95°C for 5 min, followed by 40 cycles of denaturation step at 95°C for 1 min, annealing step at 55°C for 1 min, extension step 72°C for 1 min and final extension step at 72°C for 10 min. The PCR products were visualized on 1.2% agarose gels. The PCR product of expected size was excised and purified using the QIAquick gel extraction kit (Qiagen) before ligating into pGEM^*®*^-T Easy plasmid (Promega, https://worldwide.promega.com). The ligation mixtures were transformed into *E*. *coli* JM109 competent cells (Promega) and plated on McConkey Agar/amp plates. Colonies were selected for culture and plasmid extraction using E.Z.N.A^®^ Plasmid DNA Mini Kit I (OMEGA bio-tek, http://omegabiotek.com) then subjected to DNA sequencing. The sequence of full-length *HbPR-1*cDNA was determined using the 5′/3′ RACE kit, 2^nd^ Generation (Roche, https://lifescience.roche.com) according to the manufacturer’s instructions. 3′-RACE *HbPR-1* primer (5′-TCTTGTGCATTCCAGCA ATC-3′) was designed based on the sequence of the partial PCR fragment mentioned above and the result from 3′-RACE was used to design the 5′-RACE *HbPR-1* primer (5′-ACGTGT TTTCTGTTCATTAATAAGTGAAGAAGC-3′). To confirm the assembled *HbPR-1* full-length sequence, PCR amplification was applied using specific primers designed according to the sequences of 5′- and 3′-RACE product as follows: FHbPR-1full (5′-CATCCATTGCCT AAGATCTTAAACAAACTC-3′) and RHbPR-1full (5′-ACGTGTTTTCTGTTCATTAA TAAGTGAAGAAGC-3′). The PCR reaction was performed using the EmeraldAmp^®^ PCR Master Mix (Takara, http://www.clontech.com) and carried out at 94°C for 5 min, followed by 35 cycles of 94°C for 1 min, 62°C for 1 min, 72°C for 1 min and a final extension 72°C for 10 min. The amplicon was cloned into pGEM^*®*^-T Easy plasmid (Promega) and transformed into *E*. *coli* JM109 competent cells (Promega). The positive clones were selected for plasmid extraction and DNA sequencing.

### Sequence analysis

The *PR-1* primers were designed using the PRIMER3 program (http://simgene.com/Primer3) [27]. The nucleotide and amino acid sequences were compared to those of the GenBank databases by Basic Local Alignment Search Tool (BLAST) [28] from the National Center for Biotechnology Information (NCBI) (http://blast.ncbi.nlm.nih.gov). Homologous *PR-1* gene sequences from other plant species were downloaded from NCBI and used for multiple sequence alignment using ClustalX2.1 [29]. The putative polyadenylation signal was analyzed using the Poly(A) Signal Miner program (http://dnafsminer.bic.nus.edu.sg/PolyA.html) [30]. Nucleotide translation, molecular weight and theoretical pI prediction were analyzed by ExPASy (http://web.expasy.org/compute_pi/) [31–33]. Protein domains and functional sites were scanned using ScanProsite tool (http://prosite.expasy.org/prosite.html) [34]. The putative signal peptide sequence was predicted using SignalP 4.1 server (http://www.cbs.dtu.dk/services/SignalP/) [35]. To further biological functional analysis in details, the TMHMM server (http://www.cbs.dtu.dk/services/TMHMM/) [36] and Protter server (http://wlab.ethz.ch/protter/start/) [37] were used for predicting the possible topology and localization of PR-1 protein. The prediction of protein-protein interaction sites was performed using the eFindsite server (http://brylinski.cct.lsu.edu/content/efindsiteppi-webserver) [38].

### HbPR-1 molecular structure prediction and refinement

The homology modeling method of SWISS-MODEL Workspace (http://swissmodel.expasy.org/) [39–41] and the iterative threading assembly method of I-TASSER server (http://zhanglab.ccmb.med.umich.edu/I-TASSER/) [42–44] were used for predicting the 3D structure of HbPR-1 from its amino acid sequence. The homology structural templates of HbPR-1 protein were collected from the RCSB protein databank (http://www.rcsb.org/pdb/home/) [45]. Each predicted 3D model of HbPR-1 protein was validated based on the best scoring model given by the PROCHECK tool and evaluated by ramachandran plot analysis of the ProFunc server (http://www.ebi.ac.uk/thornton-srv/databases/profunc) [46]. The PyMOL software from the BioSLAX tool (http://www.bioslax.com) [47] was used for the molecular structure visualization.

### Plasmid construction and transformation into *Agrobacterium tumefaciens*

The pGD_*HbPR-1* plasmid construction was performed by cloning PCR-amplified DNA fragment corresponding to HbPR-1 protein-encoding sequence into the binary pGD vectors [48]. The pGEM^*®*^-T Easy_HbPR-1 plasmid containing *HbPR-1* coding sequence was used as template for PCR. The oligonucleotides FPR-1_SalI (5′-gcggtcgacATGGTGTTC TGCAAGAATTC-3′) and RPR-1_BamHI (5′-gcgggatccTTA*gtggtgatggtgatggtg*ATAAGG TTTCTGCCCAAC-3′) were used for fragment amplification. The introduced SalI and BamHI restriction sites were underlined. The italic letters represent the hexahistidine-tag sequences. The PCR was performed using the Phusion^®^ HF DNA polymerase (New England Biolabs Inc., https://www.neb.com) and was carried out at 98°C for 30 s, followed by 35 cycles of 98°C for 15 s, 60°C for 15 s, 72°C for 1 min and a final extension 72°C for 10 min. The amplified fragment was cloned into SalI and BamHI sites of pGD [48]. The pGD_HbPR-1 plasmid containing *HbPR-1* gene insert with the correct nucleotide sequences was used to electroporate into *A*. *tumefaciens* C58-C1.

### Transient expression of HbPR-1 in *N*. *benthamiana*

Transient expression of HbPR-1 in *N*. *benthamiana* was conducted according to the agroinfiltration method described by Kruger et al. [49]. *A*. *tumefaciens strain* C58-C1 carrying the pGD_HbPR-1 plasmid and *A*. *tumefaciens strain* GV3101 carrying the pJL3-p19 plasmid [50] were grown in Luria-Bertani media supplemented with appropriate antibiotics at 28°C. Overnight agrobacterial cultures were collected from plates and re-suspended in infiltration/induction media (10 mM MgCl_2_, 10 mM MES, pH 5.6, and 150 μM acetosyringone). The *A*. *tumefaciens* cultures of pGD_HbPR-1 or pJL3-p19 were prepared with an optical density (OD600) of 0.4 for infiltration of single strain. For co-infiltration, two cultures were prepared with OD600 of 0.8 and then mixed with equal volumes of each one. The mixtures were kept for 3 h at room temperature. The infiltration/induction media buffer (mock, negative control) or the mixtures were infiltrated into 6-week-old leaves of *N*. *benthamiana* plants using 1 mL needleless syringe to the abaxial surface of fully expanded leaves. Analysis of PR-1 protein expression and isolation of intercellular fluids were conducted at 48 h after infiltration.

### Analysis of PR-1 protein expression in *N*. *benthamiana*

PR-1 protein expression in *N*. *benthamiana* was evaluated by Western blot analysis. Total proteins were obtained as described: leaf discs were collected using No.7 cork borer and transferred into 2-mL screw cap tube. The sample was disrupted by bead homogenizer method using grinding balls and a FastPrep^®^-24 homogenizer (MP Biomedicals). The homogenates were extracted with 2x Laemmli buffer [51], boiled for 5 min and centrifuged at 13,000 rpm for 5 min. The supernatant was loaded onto 15% sodium dodecyl sulfate polyacrylamide gel electrophoresis (SDS–PAGE) and analyzed by Western blot.

### Isolation of intercellular fluids

Intercellular fluids were collected from *N*. *benthamiana* leaves co-infiltrated with *A*. *tumefaciens* C58-C1 (pGD_HbPR-1) and *A*. *tumefaciens* GV3101 (pJL3-p19) or infiltrated with *A*. *tumefaciens* GV3101 (pJL3-p19) only using an extraction buffer (300 mM NaCl, 50 mM NaPO4, pH 7) according to the method described previously [52]. The intercellular fluids were filter-sterilized and analyzed by 15% SDS–PAGE and Western blot analysis.

### Purification of the PR-1 protein

The intercellular fluid containing the hexahistidine-tagged HbPR-1 was used for purification of HbPR-1 protein expressed in *N*. *benthamiana* plants. NaCl and imidazole stock solutions were slowly added to 20-mL of intercellular fluid to a final concentration of 500 mM NaCl and 20 mM imidazole. The mixture was loaded into 2 mL of the Complete his-tag purification resin (Roche) that was pre-equilibrated with wash buffer (25 mM HEPES, pH 7.5, 500 mM NaCl, 20 mM imidazole, 10% glycerol). After washing with wash buffer, the bound proteins were eluted with multiple 1 mL of elution buffer (wash buffer with different concentrations of 50–250 mM imidazole). 1 mL fractions were collected and analyzed by 15% SDS-PAGE to determine the presence of the hexahistidine-tagged HbPR-1. The fractions containing the purified proteins were pooled and then desalted and concentrated using Amicon^®^ultra-15 Centrifugal Filters (Merck, http://www.merckmillipore.com) according to the manufacturer’s protocol. The protein concentration was determined using the Bradford protein assay kit (Bio-Rad, http://www.bio-rad.com).

### SDS-PAGE and Western blot analysis

The obtained proteins were analyzed by 15% SDS-PAGE and stained with Coomassie Brilliant Blue as previously described by Sambrook et al.[53] or the proteins were transferred to a polyvinylidenedifluoride (PVDF) membrane (Thermoscientific, https://www.thermofisher.com) for Western blot. HRP conjugated anti-His monoclonal antibody His-probe (H-3) (sc-8036 HRP, Santa Cruz Biotechnology, INC., http://www.scbt.com) was used for the detection of hexahistidine-tagged HbPR-1 proteins, which were visualized with 1-Step Ultra TMB-Blotting Solution (Thermoscientific). The size of proteins was estimated with Precision plus protein^™^ all blue standards (10–250 kDa, BIO-RAD).

### The role of PR-1 in the protection of plant against the pathogen

The role of PR-1 protein in plant defense response against the pathogen was conducted using *N*. *benthamiana* leaves expressing HbPR-1 by *Agrobacterium*-mediated transient expression. The leaves were infiltrated with *Agrobacterium* carrying *HbPR-1* gene and then inoculated with zoospores of the oomycete pathogen *P*. *palmivora*. The leaf was divided into two halves based on the midvein. The first half was co-infiltrated with *A*. *tumefaciens strain* C58-C1 carrying the pGD_HbPR-1 plasmids and *A*. *tumefaciens strain* GV3101 carrying the pJL3-p19 plasmid. The other half which served as control was infiltrated only with *A*. *tumefaciens strain* GV3101 carrying the pJL3-p19 plasmid. After 24 h of infiltration, 10-uL of 5×10^2^ zoospore/mL or 1×10^3^ zoospore/mL was inoculated on 90 leaves from 30 different plants (for each zoospore suspension concentration). Disease incidence and lesion diameter on each leaf were recorded on 3, 4 and 5 d after inoculation.

### Effect of recombinant PR-1 protein on *P*. *palmivola* zoospore germination

The effect of the PR-1 protein on inhibition of *P*. *palmivora* zoospore germination was evaluated using sterile distilled water as negative control and antibiotic G418 as positive control. Zoospore suspension (2×10^5^ zoospores/mL) was prepared in sterile distilled water from 5-day-old cultures of *P*. *palmivora*. Fifty microlitters of this zoospore suspension were added to equal volume of purified HbPR-1 (200 μg/mL), G418 antibiotic (200 μg/mL) or sterile distilled water. After 30 min, 10 μL of each mixture were dropped on 1.5% water agar plates which were then incubated at room temperature for 2 h. Germinated zoospores were counted under an optical microscope and the germination rates were calculated. The experiment was performed with three replicates.

### Statistical analysis

Statistical analysis was performed by one-way analysis of variance (ANOVA) with Duncan’s multiple range test using SPSS Statistics 17.0 software. Statistical significance was taken at P<0.05. All results were presented as the means±SE.

## Results

### Isolation and molecular characterization of *HbPR-1*

The *HbPR-1* (*PR-1* of *Hevea brasiliensis* or rubber tree) cDNA was successfully isolated from 24 h SA-treated rubber tree leaves using RT-PCR and RACE techniques. The full-length cDNA of *HbPR-1* had a total length of 647 nucleotides containing a 34 bp of 5′-untranslated region (UTR) located upstream of a start codon (ATG) and 121 bp of 3′-UTR that ended with a poly (A) tail. The putative polyadenylation signal sequences, ATAAA, were located downstream of a stop codon (TAA) at nucleotide position 606. The ORF was 492 bp which corresponds to a predicted translated product of 163 amino acids (S1 Fig). The complete cDNA sequence of *HbPR-1* gene has been submitted to the GenBank with an accession number KM514666.

**Fig 1:**
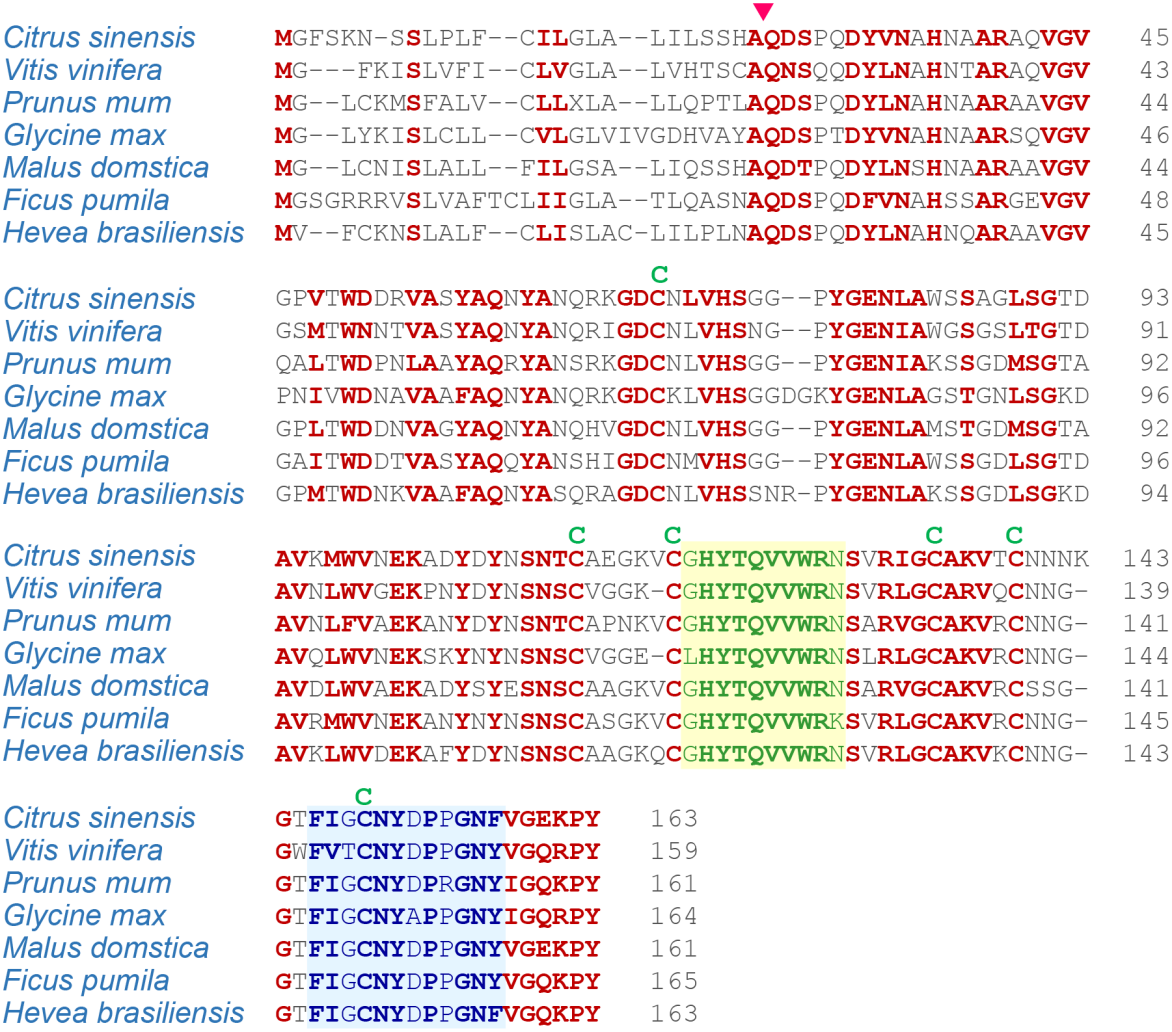
Amino acid sequence alignment of the HbPR-1 from *H*. *brasiliensis* with its homologous proteins of different plant species. The sequences of the plant PR-1 proteins were obtained from the GenBank database: *Citrus sinensis* (XP_006486822.1), *Vitis vinifera* (XP_ 002273416.1), *Prunus mum* (XP_008236225.1), *Glycine max* (XP_003545771.1), *Malus domstica* (XP_008370577.1), *Ficus pumila var*. *awkeotsang* (AFK93500.1). The sequences were aligned by Clustal-X (Thompson et al., 2001). The conserved amino acid sequences were highlighted in red which indicates 100% conserved sequences. The arrowhead indicated the cleavage site between the signal peptide and the mature protein. The positions of the cysteine residues forming disulfide linkages were shown as C. CRISP family signature 1 (CRISP_1) were highlighted in green and CRISP family signature 2 (CRISP_2) were highlighted in blue.

**Fig 2:**
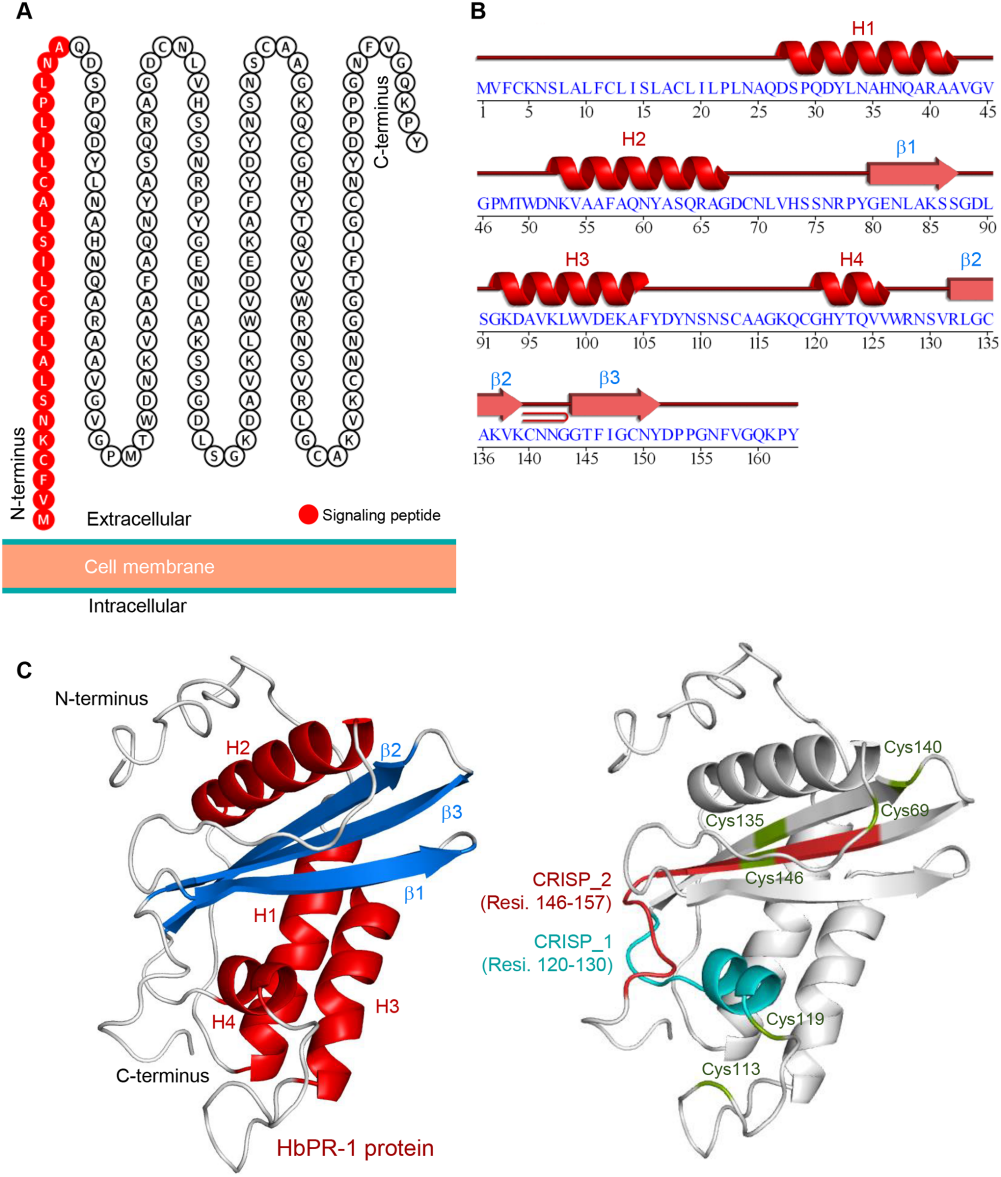
The putative cellular localization and predicted structure of HbPR-1. (A) HbPR-1 protein localization predicted by the Protter server. The result suggested that the HbPR-1 protein was located at the extracellular side of the cell membrane. The twenty-five amino acids (red color) at the N-terminus represent the predicted signaling peptide. (B-C) The cartoon representation of the model of HbPR-1 protein predicted using the SWISS-MODEL and I-TASSER server. (B) The graphical display of the 2D topology of the predicted HbPR-1 model. (C) The cartoon structure representation of HbPR-1 3D model with the four α-helices, three β-sheets, seven strands and one junction loop.

The deduced amino acids of HbPR-1 were predicted to have a molecular mass of 17,681 Da and an isoelectric point (pI) of 8.56. HbPR-1 possessed a hydrophobic signal peptide as predicted using SignalP4.1 program. The predicted cleavage site of signal peptide and mature protein was between amino acid A (Alanine) and amino acid Q (Glutamine) (Fig 1). Moreover, by using the primary amino acid sequence of HbPR-1 protein, a high score from the TMHMM result indicated that 163 amino acids of HbPR-1 protein were located on the outside of the cell membrane and the amino acid residues Met1-Ala25 matched the signal peptide with probability score of 0.70. The Protter results also suggested that HbPR-1 protein is located at the extracellular side of the cell membrane. Moreover, Protter tool showed that the twenty-five amino acid residues on the N-terminus end of PR-1 protein have the potential as a signal peptide (Fig 2A). All above analyses suggest that the HbPR-1 protein is secreted into the extracellular space in leaves of rubber tree.

The mature HbPR-1 protein contained six conserved cysteine residues (Fig 1). BLASTP search revealed that HbPR-1 amino acid sequences had the conserved domain of SCP (sperm coating protein)-PR-1_like: SCP-like extracellular protein domain. SCP-like extracellular protein domain may act as an antifungal agent or be involved in cell wall loosening. This family also includes CRISPs (cysteine rich secretory proteins) which combine SCP with a C-terminal cysteine rich domain [54]. Corresponding to the result from ScanProsite tool analysis, HbPR-1 protein contained two domains; CRISP family signature 1 (CRISP_1) at position 120–130 (GHYTQVVWRNS) and CRISP family signature 2 (CRISP_2) at position 146–157 (FIGCNYDPPGNF) (Fig 2).

The deduced amino acid sequences of *HbPR-1* were compared to the *PR-1* genes of different plant species (*Citrus sinensis*, *Vitis vinifera*, *Prunus mum*, *Glycine max*, *Malus domstica*, *Ficus pumila var*. *awkeotsang*) using the CLUSTALX program (Fig 1). Similarity searches of the HbPR-1 protein against the non-redundant database of GenBank^™^ were conducted using the BLASTP program. The amino acid sequences of HbPR-1 have close sequence identity to plant PR-1 proteins in both monocots and dicots (Fig 1).

### Homology modeling and analysis of HbPR-1 protein

The predicted 3D model of HbPR-1 protein was simulated using SWISS-MODEL and I-TASSER server (Fig 2B and 2C). The homologous template structures of HbPR-1 protein were collected from the RSCB databank, including the NMR solution structure of the chain A of *Solanum lycopersicum* pathogenesis-related protein P14a, the x-ray diffraction structure of *Vespula vulgaris* pathogenesis-related protein superfamily, human Golgi-associated plant pathogenesis-related protein and human Glioma pathogenesis-related protein 1 (PDB ID: 1CFE, 1QNX, 1SMB and 3Q2U, respectively). The predicted 3D structure of HbPR-1 was composed of four α-helices, three β-sheets, seven strands, and one junction loop (Fig 2B and 2C). To assess the quality of the predicted model of HbPR-1, the Ramachandran plot analysis was used to visualize backbone dihedral angles ψ against φ of amino acid residues that reflects the stability of the HbPR-1 structure prediction. The amino acid analysis result for HbPR-1 structure prediction showed 98.60% residues plotted in the allowed regions, suggesting that the HbPR-1 structure prediction has good stability (S2 Fig).

According to the predicted 3D structure of the HbPR-1 protein (Fig 2), the pairs of cysteine residues that form the disulfide bridges were identified as Cys69/Cys140, Cys113/Cys119 and Cys135/Cys146, respectively. Moreover, the CRISP family signature 1 (amino acid residues at position 120–130) and CRISP family signature 2 (amino acid residues at position 146–157) were also identified (Fig 2C).

**Fig 3:**
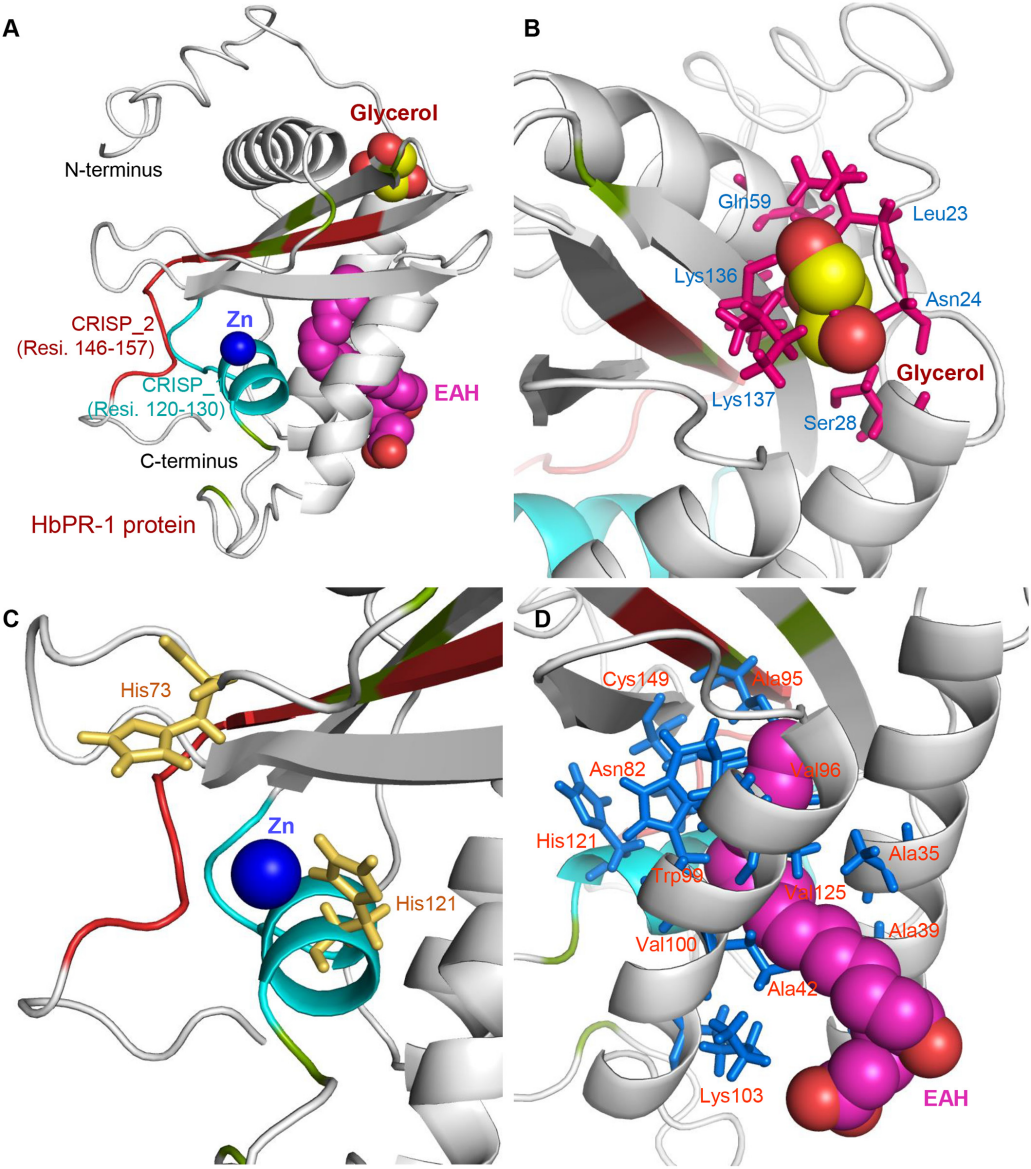
The putative binding sites of HbPR-1 protein. (A) The putative binding sites of HbPR-1 protein were predicted by the I-TASSER. The result suggested the possible locations of HbPR-1 protein interacting with glycerol (red-yellow color), Zn^2+^ (navy blue color) and EAH (pink-red color). (B-D) The putative residues of HbPR-1 protein that might be bound to the glycerol molecule (B), Zn^2+^ ion (C) and EAH molecule (D), respectively.

For further investigation of HbPR-1 amino acids within the 3D predicted structure, the potential binding sites were predicted using I-TASSER server. The I-TASSER prediction results suggested that the HbPR-1 structure had the important putative sites that could bind three molecules: Glycerol, Zn^2+^ and EAH (5S,7E,9E,11Z,14Z)-5-hydroxyicosa-7,9,11,14-tetraenoic acid) (Fig 3). With the prediction sites of the HbPR-1 model, the amino acid residue Leu23, Asn24, Ser28, Gln59 and Lys136-137 showed a possible interaction with Glycerol (Fig 3B). The His73 residue on CRISP_1 domain and His121 of HbPR-1 could bind Zn^2+^ ion (Fig 3C). Amino acid residue Ala35, Ala39, Ala42, Asn82, Ala95, Val96, Trp99, Val100, Lys103, His121 and Cys142 within α-helix H1 and H3 were possibly bound to EAH molecule (Fig 3D). To further analyze HbPR-1 structure in details, we have predicted the protein-protein interaction sites by using the eFindSite server. The eFindSite prediction result suggested that HbPR-1 had a high confidential score of protein-protein binding with the thirteen interfacial residues: Asn70, Val72-Asn76, Gln118, Gly120, Asn156-Phe157, and Gly159-Lys161, respectively. Furthermore, the eFindSite prediction hinted that the Asn156 residue was the hydrogen bond binding and the His73 residue was aromatic interaction site.

#### Expression of recombinant HbPR-1 in *N*. *benthamiana*

**Fig 4:**
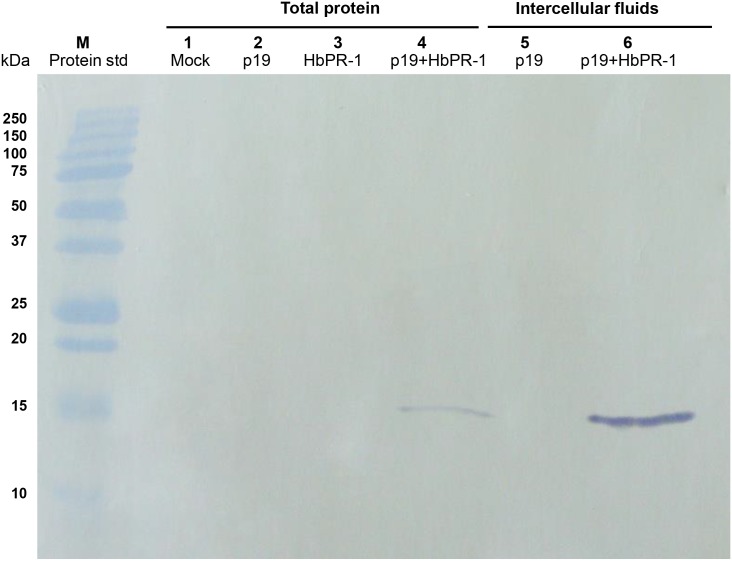
Transient expression of HbPR-1 protein in *N*. *benthamiana*. Total proteins and intercellular fluids were isolated from agroinfiltrated *N*. *benthamiana* plants and were visualized by Western blot analysis. Lane M represents protein standard and lanes 1–4 represent total proteins isolated from infiltrated *N*. *benthamiana* leaves with infiltration buffer (Mock, lane 1), *A*. *tumefaciens* GV3101 expressing pJL3-p19 (lane 2), *A*. *tumefaciens* C58C1 expressing pGD_HbPR-1 (lane 3) and a mixture of *A*. *tumefaciens* strains expressing pJL3-p19 and pGD_HbPR-1 (lane 4). Lane 5 and 6 represent intercellular fluids isolated from infiltrated *N*. *benthamiana* leaves with *A*. *tumefaciens* GV3101 expressing pJL3-p19 (lane 5) and a mixture of *A*. *tumefaciens* strains expressing pJL3-p19 and pGD_HbPR-1 (lane 6), respectively. The numbers on the left represent the size of molecular weight markers.

In order to investigate the function of *HbPR-1* gene, agroinfiltration was used for transient expression of HbPR-1 fused with the hexahistidine-tag at the amino terminus in *N*. *benthamiana* leaves. Total proteins and intercellular fluids were extracted from leaves infiltrated with single strain or a combination of *A*. *tumefaciens* cultures expressing pJL3-p19 or pGD_HbPR-1. Recombinant HbPR-1 protein was detected by Western blot with HRP conjugated anti-His monoclonal antibody. A single band which corresponds to molecular weight of the expected HbPR-1 protein (17 kDa) was observed in samples from leaves co-infiltrated with *A*. *tumefaciens* strains expressing pJL3-p19 and pGD_HbPR-1 (Fig 4). However, the band was not detected in the samples from leaves infiltrated only with *A*. *tumefaciens* expressing pJL3-p19 and the mock control, suggesting that this band represents HbPR-1 protein (Fig 4). The HbPR-1 protein from single infiltration was barely detected and therefore much less than that from co-expression of HbPR-1 and p19, indicating that *Agrobacterium*-mediated transient expression was enhanced by p19 gene silencing suppressor protein. In addition, the band was observed from intercellular fluids indicating that the recombinant HbPR-1 protein was an extracellular protein, which is consistent with the predicted results using Protter and TMHMM program that the HbPR-1 protein was located at the extracellular side of the cell membrane. The above results demonstrated that expression of HbPR-1 by agroinfiltration was successful, which promised successful purification of HbPR-1 from isolated intercellular fluid.

#### Purification of recombinant HbPR-1 protein expressed in *N*. *benthamiana*

To investigate whether the recombinant HbPR-1 protein expressed in the extracellular space of *N*. *benthamiana* can be purified by affinity chromatography, the intercellular fluids isolated from *N*. *benthamiana* leaves co-expressing p19 and HbPR-1 proteins were applied onto a column packed with Complete his-tag resin. The bound HbPR-1 was eluted from the column by elution buffer containing different concentrations of 50–250 mM imidazole. The fractions containing HbPR-1 were pooled, desalted and concentrated.

**Fig 5:**
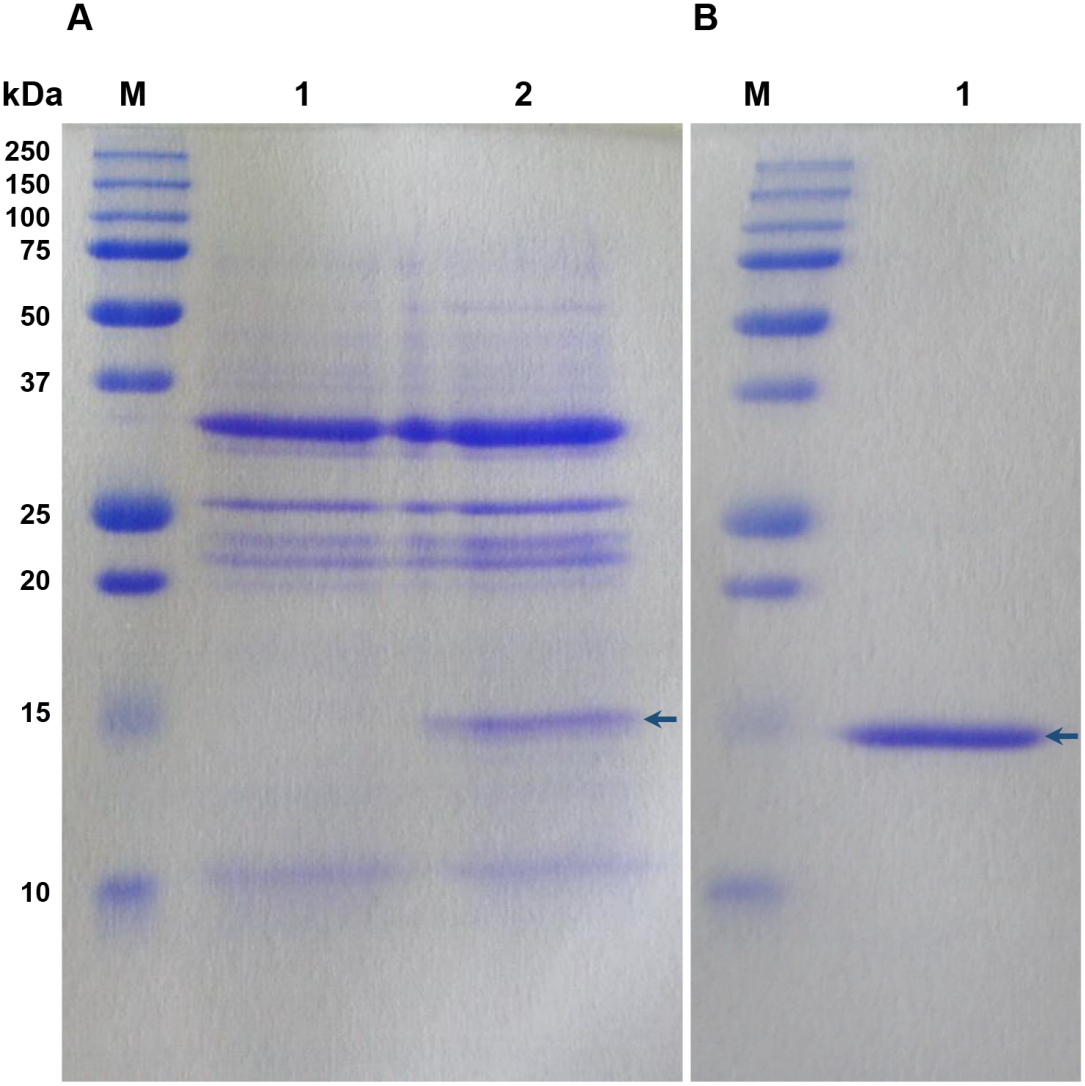
Purification of HbPR-1 protein expressed in the extracellular space of *N*. *benthamiana* leaves. (A) SDS-PAGE of the intercellular fluids isolated from *N*. *benthamiana* leaves. Lane 1 indicates intercellular fluid isolated from *N*. *benthamiana* leaves infiltrated with *A*. *tumefaciens strain* GV3101 carrying the pJL3-p19 and lane 2 indicates intercellular fluid isolated from *N*. *benthamiana* leaves co-infiltrated with *A*. *tumefaciens* C58-C1 carrying the pGD_HbPR-1 and *A*. *tumefaciens* GV3101 carrying the pJL3-P19. (B) SDS-PAGE of the purified HbPR-1 protein. The protein was purified from intercellular fluid shown in A (lane 2) by affinity chromatography with complete his-tag resin. Lane M indicates protein standard and the numbers on the left represent the size of molecular weight markers.

The intercellular fluids before purification (Fig 5A) and the purified protein (Fig 5B) were run on SDS-PAGE and stained with Coomassie Brilliant Blue. The purified HbPR-1 protein appeared as a single band with an apparent molecular mass of approximately 17 kDa (Fig 5B), the same size as the distinct band which was only present in intercellular fluids collected from leaves co-expressing p19 and HbPR-1 but not in the control (Fig 5A). HbPR-1 was purified to a high purity after one-step purification process with affinity column, indicating that the system established for HbPR-1 purification was very efficient.

### Role of HbPR-1 in plant defense

**Fig 6:**
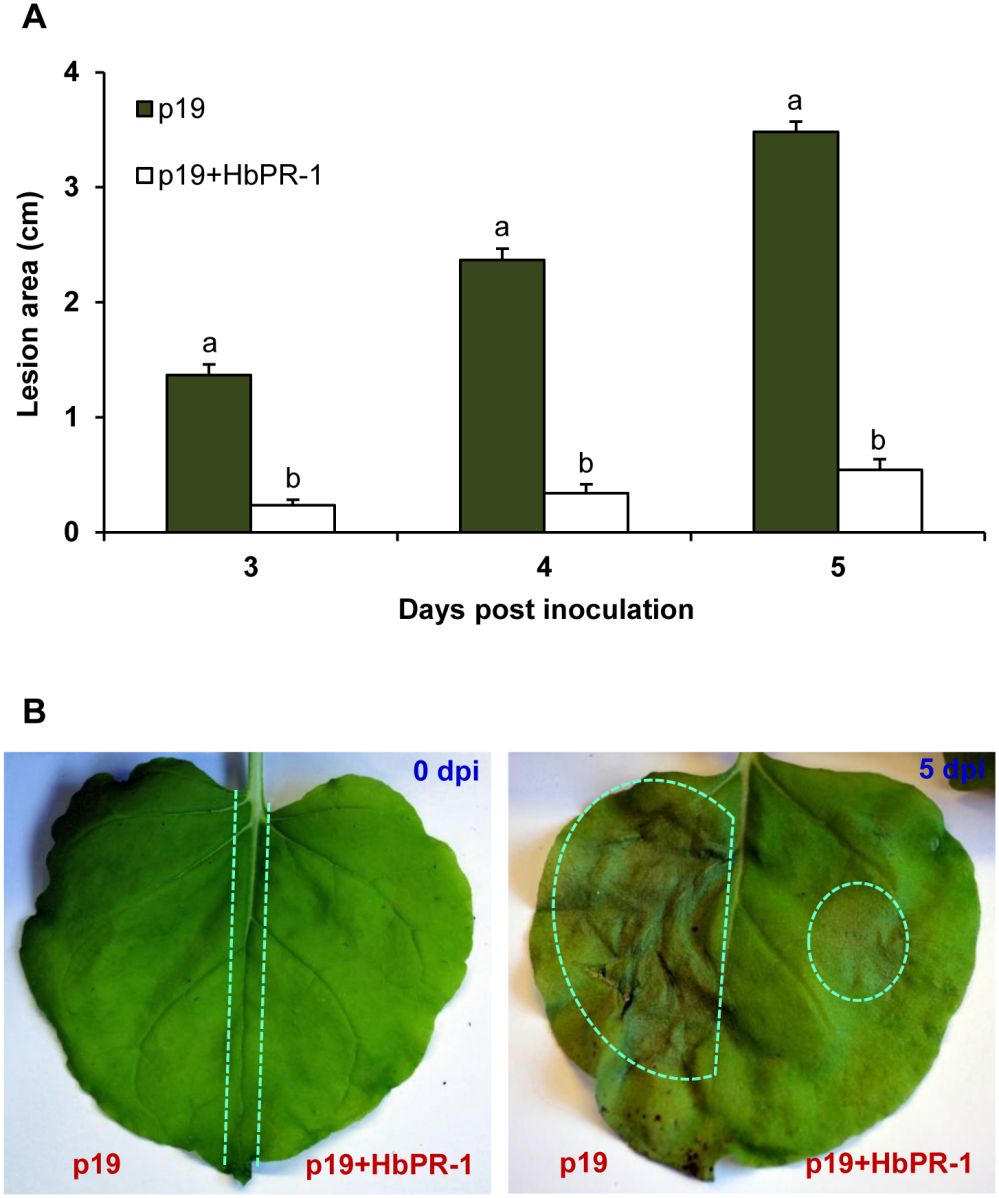
Effect of HbPR-1 on the protection of *N*. *benthamiana* against *P*. *palmivora*. Half leaf was infiltrated with *A*. *tumefaciens* GV3101 carrying the pJL3-p19 (p19) and the other half was infiltrated with *A*. *tumefaciens* C58-C1 carrying the pGD_HbPR-1 and *A*. *tumefaciens* GV3101 carrying the pJL3-p19 (p19+HbPR-1). After 24 h, the leaves were inoculated with 10-μL of 1×10^3^ zoospores/mL. Data represented the average diameter of lesion area with standard error from 90 different leaves of 30 plants. Bars with different letters within day indicated statistically significant differences at P<0.05 according to the Duncan’s multiple range test. (A) Average lesion area of *N*. *benthamiana* after inoculation with 1×10^3^ zoospores/mL *P*. *palmivora* on 3, 4 and 5 days post inoculation (dpi). (B) Photographs of representative *N*. *benthamiana* leaves expressing p19 or p19 together with HbPR-1 at 0 and 5 dpi.

To evaluate the effect of overexpression of *HbPR-1* genes on generation of *N*. *benthamiana* resistance to the oomycete pathogen *P*. *palmivora*, *N*. *benthamiana* leaves were infiltrated with *A*. *tumefaciens* cultures of pJL3-p19 (negative control) or co-infiltrated with *A*. *tumefaciens* cultures of pJL3-p19 and pGD_HbPR-1. The infiltrated leaves were then inoculated with 10-μL of 5×10^2^ zoospore/mL or 1×10^3^ zoospore/mL of *P*. *palmivora*. The localized necrosis area was observed after inoculation. The expression of HbPR-1 significantly reduced the localized necrosis area (Fig 6). When inoculated with 5×10^2^ zoospores/mL, the expression of HbPR-1 reduced necrosis areas by 94.4, 92.0 and 87.6% on 3, 4 and 5 dpi respectively compared to control. Similar reduction of necrosis areas were also observed when inoculated with 1×10^3^ zoospores/mL, with reductions of 83.0, 85.6 and 84.5%, respectively (Fig 6A). These results indicated that the expressed HbPR-1 protein played an important role in *N*. *benthamiana* resistance to *P*. *palmivora*.

### Inhibition of HbPR-1 protein on *P*. *palmivora* zoospore germination

**Fig 7:**
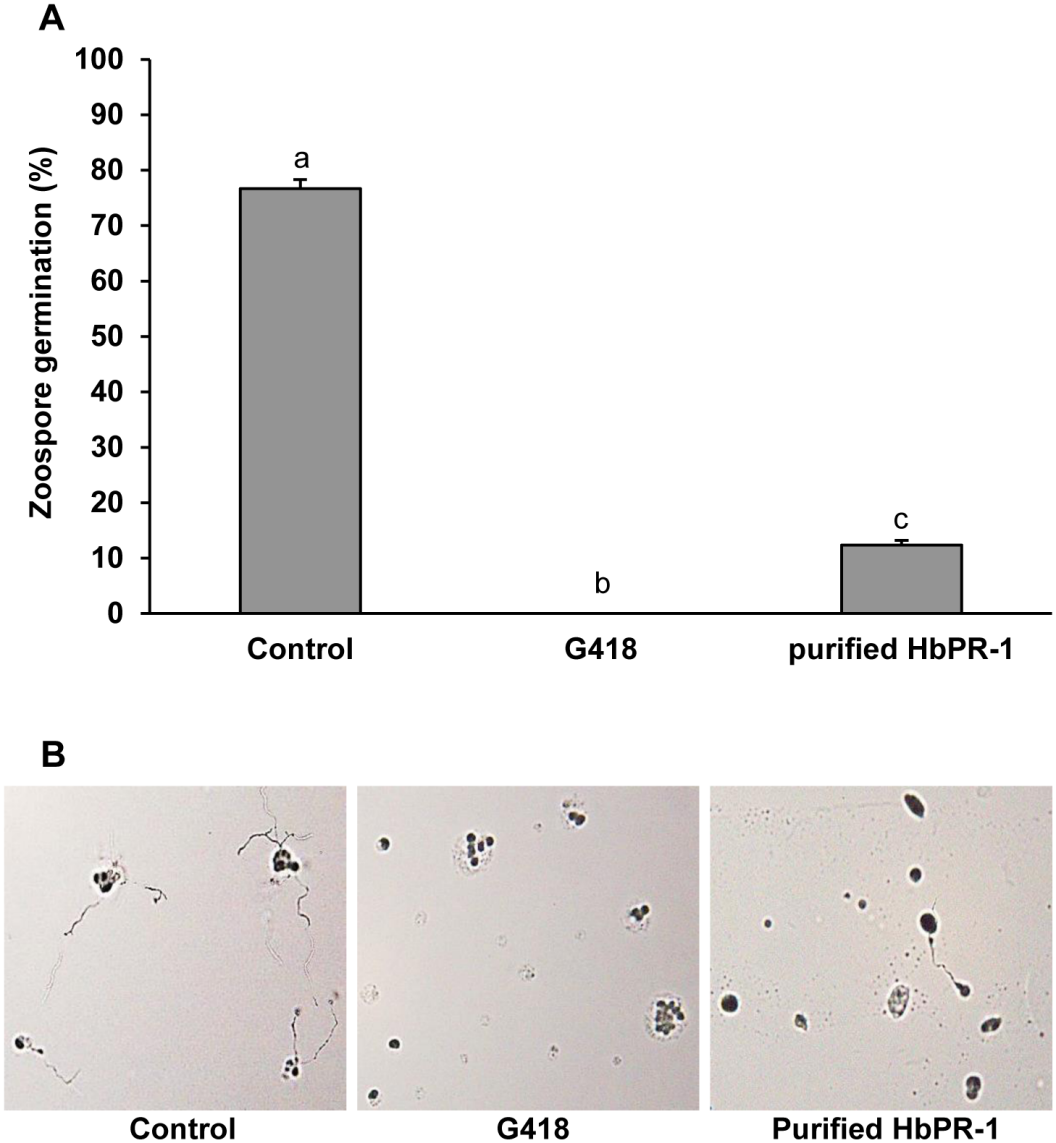
Effect of purified HbPR-1 on germination of *P*. *palmivora* zoospores. **(A)** The percentage of zoospore germination after zoospores were treated with purified PR-1 or sterile distilled water (control) or G418 for 30 min and then grown on 1.5% water agar for 2 h. Data represent the average of percentage of zoospore germination with standard error of three replicates. Bars with different letters indicated statistically significant differences at P<0.05. (B) Photographs of zoospores of *P*. *palmivora* grown on 1.5% water agar after treatments.

To determine the function of recombinant HbPR-1 protein on inhibiting *P*. *palmivora* zoospore germination, zoospores of *P*. *palmivora* were incubated with purified HbPR-1 protein for 30 min and then grown on a water agar plate for 2 h. The zoospore germination was observed and compared with the one in sterile distilled water and antibiotic G418. HbPR-1 exhibited strong inhibition of *P*. *palmivora* zoospore germination compared with the negative control (sterile distilled water) whereas G418 showed complete inhibition (Fig 7). The recombinant HbPR-1 inhibited approximately 64% of zoospore germination compared with control. This result indicated that recombinant HbPR-1 protein was an efficient antimicrobial protein against *P*. *palmivora*.

## Discussion

The full-length cDNA of *HbPR-1* had a total length of 647 nucleotides which were predicted to be translated to a product of 163 amino acids with a molecular mass of 17,681 Da and an isoelectric point (pI) of 8.56. Based on the isoelectric point (pI), the PR-1 proteins were classified into two groups as acidic and basic forms [7]. Previously, two rice *PR-1* genes were identified to encode an acidic PR1a protein (pI 4.4) and a basic PR1b protein (pI 8.0) [11]. In addition, an acidic PR-1 protein (pI 5.14) was identified from *Oryza grandiglumis* leaves [15]. Here we characterized HbPR-1 protein as belonging to the basic PR-1 type.

The mature HbPR-1 protein contained six conserved cysteine residues and two CRISPs (cysteine rich secretory proteins) domains. Generally, mature PR-1 proteins contain six conserved cysteine residues which form disulfide bridges, and exhibit high sequence conservation level in all plant families [7]. CRISPs are thought to be involved in the plant defense against pathogens and plant stress resistance [55–56]. Since the amino acid sequence of domains in PR-1 proteins showed high conservation in plants, Van Loon and Van Strien [7] suggested that these domains play an important role in the PR-1 protein function.

The 3D structure of HbPR-1 predicted in this study was composed of four α-helices, three β-sheets, seven strands and one junction loop. This was in accordance with the PR-1-type proteins which generally have a specific structure with four α-helices and several β-sheets arranged in antiparallel between helices [6–7,57]. Moreover, all PR-1 proteins also contain six cysteines, ones of a number of conserved residues found in these proteins. These findings indicate that a protein module having been preserved during evolution is defined by the unique molecular structure of PR-1 which must serve important functions [10].

According to the potential binding sites of the predicted structure, the HbPR-1 had the important putative sites that could bind three molecules: Glycerol, Zn^2+^ and EAH (5S,7E,9E,11Z,14Z)-5-hydroxyicosa-7,9,11,14-tetraenoic acid). Amino acid residues showed a possible interaction with fatty acid (EAH) and glycerol which is a component of fat molecules, suggesting that amino acid residues of HbPR-1 protein could bind lipid molecules which are components of microbial cell wall. Park et al. [58] found that purified PR-1 protein from pumpkin rind inhibited growth of various fungal pathogens and had membrane permeabilization activity. The damage of the fungal cellular membrane is directly induced by PR-1, resulting in a leakage of cytoplasmic components to the exterior of cell. Therefore, the prediction results in this work could lead to a further study on the PR-1-lipid interaction in order to understand the role of PR-1 in the antimicrobial activity and plant defense. Moreover, the His73 residue on CRISP_1 domain and His121 of HbPR-1 could bind Zn^2+^. CRISPs which have been identified as a toxin family in most animal venoms can bind Zn^2+^ at their N-terminal PR-1 domain, but their function remains unknown [59]. Zinc coordination environments in proteins have been defined as catalysis, cocatalysis, structure and interface [60]. Thus, it is probable that PR-1 in plants binds Zn^2+^ in order to obtain correct folding of the polypeptide chain. PR-1 may also bind other molecules containing Zn^2+^, which involves in plant defense.

To date, much of the information about the roles and biochemical functions of PR-1 is still unknown. However, it has been reported that PR-1 enhances plant resistance to various pathogens.

Ectopic expression of a pepper basic pathogenesis-related protein 1 gene (*CABPR1*) in tobacco plants (*N*. *tabacum* cv. xanthi) enhanced resistance to the pathogens *Ralstonia solanacearum*, *Pseudomonas syringae* pv. *tabaci* and *P*. *nicotianae* [1]. Transgenic tobacco plants overexpressing a basic-type PR-1 gene showed significantly greater antibacterial resistance to *P*. *syringae* pv. *tabaci* than when overexpressing an acidic-type PR-1 gene or control [61]. The overexpression of a pepper basic PR protein 1 gene in tobacco plants may activate a signal transduction pathway that may be related to the resistance against pathogen disease. It may involve reactive oxygen species (ROS) or ion flux generation that causes the activation of defense mechanisms [1]. Although the key mechanisms are still unknown, a role for PR-1 in programmed cell-death (PCD)-related pathways was supported by the fact that PR-1 proteins are pathogen-inducible and frequently isolated from HR-associated plant tissues [9]. Moreover, HbPR-1 protein is an extracellular protein that can effectively prevent pathogen invasions. Although the extracellular defense-related proteins are in a location that they can contact invading pathogens before the tissue has been penetrated, it takes time before the proteins start accumulation. Therefore, effective pathogens’ passing into further tissues is likely to occur before the induced proteins become sufficiently active. As a result, the function of these proteins may be effective against following invaders, or involve the development of SAR which provides biochemical barrier against subsequent infections [10].

The recombinant HbPR-1 protein was found to be an antimicrobial protein against *P*. *palmivora*, which is in agreement with previous reports showing that PR-1 proteins exhibited a broad-spectrum antimicrobial activity. Zhu *et al*. [6] expressed and purified *N*. *benthamiana* PR-1 (NbPR-1) possessing antimicrobial activity against multiple phytopathogenic fungi including *Bipolaris maydis*, *Fusarium graminearum*, *Aspergillus oryzae*, *A*. *niger* and *Sclerotinia sclerotiorum*. The recombinant *Nepenthes mirabilis* PR-1 (NmPR-1) protein was found to confer antibacterial activity against both gram-negative *P*. *syringae* pv. *glycenia* and *Escherichia coli* and gram-positive bacteria *Bacillus subtilis* [57]. Moreover, purified PR-1 protein from pumpkin rind has antifungal activity against *F*. *moniliforme*, *F*. *oxysporum*, *F*. *solani*, *Collectrichum coccodes*, *Botryris cinerea*, *Rhizoctonia solani*, *Candida albicans* and *A*. *flavus*. PR-1 is thought to damage the fungal cellular membrane and inhibit fungal growth through osmosis or efflux of intracellular components [58]. It has been reported that PR-1 of tobacco and tomato exhibits anti-oomycete activity [1]. Similarly, the obtained results from this study showed that HbPR-1 had an activity against oomycete *P*. *palmivora*. The inhibitory effects of plant PR-1 on many pathogens imply that HbPR-1 may also inhibit other pathogens in addition to *P*. *palmivora*.

## Supporting Information

S1 Fig Nucleotide and deduced amino acid sequences of *HbPR-1* gene identified from the leaves of *H*. *brasiliensis*. The nucleotide sequence is shown in black with the letters in upper case representing protein-encoding sequence and the letters in lower case representing untranslated regions. An asterisk (_*_) represents the stop codon. The underlined letters showed the putative polyadenylation signal (aataaa). The deduced amino acid sequence is shown with blue letters. The sequence was submitted to GenBank with accession number KM514666.
(TIF)

S2 Fig Ramachandran analysis of the PR-1 model from *H*. *brasiliensis*. The graphic shows that 72.1% (101 residues) of HbPR-1 amino acids was plotted in the most favored regions, 23.6% (33 residues) in additional allowed regions, 2.9% (4 residues) in generously allowed regions and 1.4% (2 residues) in disallowed regions, respectively. The 85.9% (140 residues) was plotted in non-glycine and non-proline residues, 1.2% (2 residues) represented in the end-residues (excl. Gly and Pro), 8.6% (14 residues) was the glycine residues and 4.3% (7 residues) was the proline residues.
(TIF)
