# Supplementary figures and images for: Molecular Cloning of HbPR-1 Gene from Rubber Tree, Expression of HbPR-1 Gene in Nicotiana benthamiana and Its Inhibition of Phytophthora palmivora
Source: PLoS One. 2016 Jun 23;11(6):e0157591. doi: 10.1371/journal.pone.0157591 (PMC4940168; doi:10.1371/journal.pone.0157591)

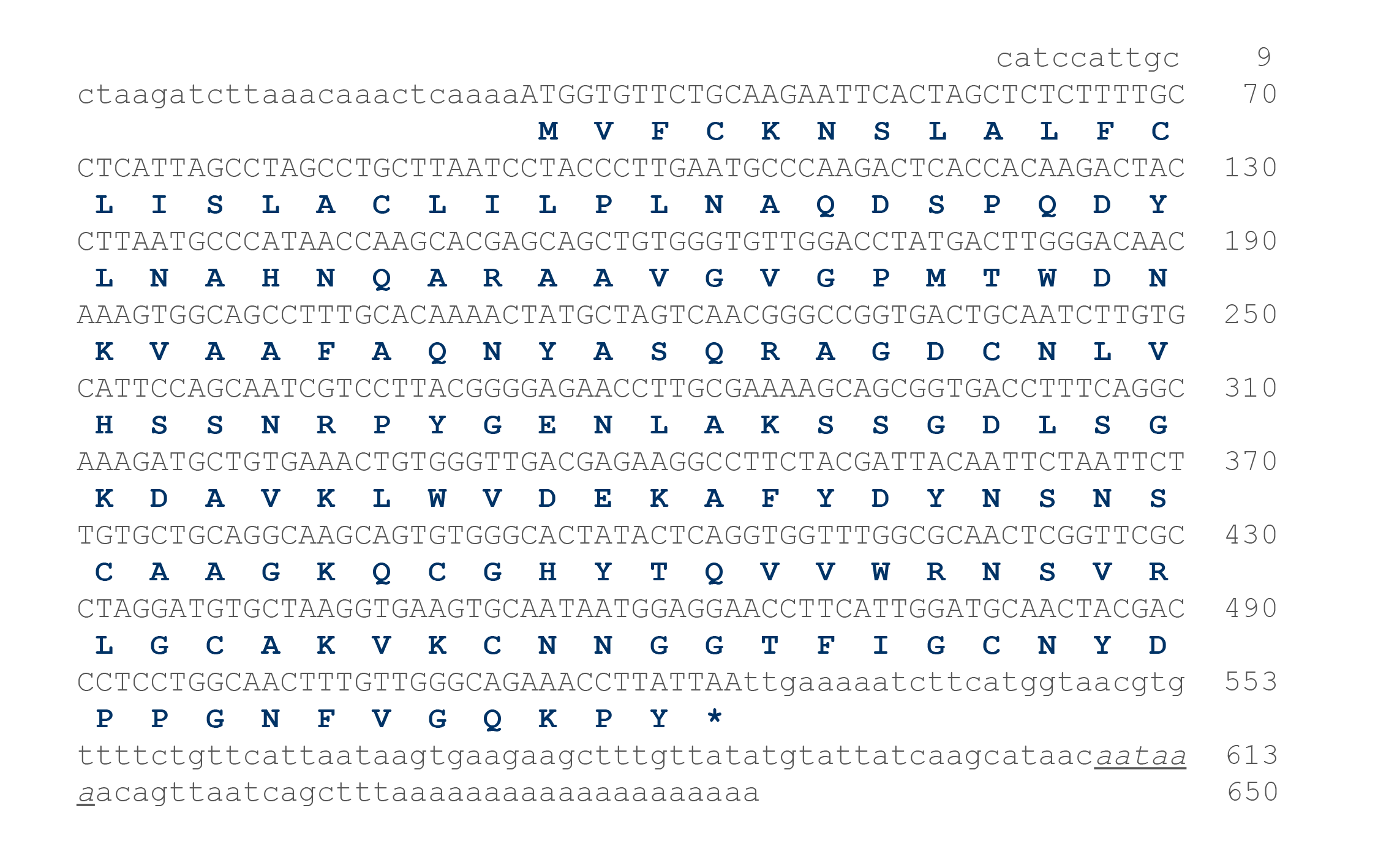

Supplement: S1 Fig — The nucleotide sequence is shown in black with the letters in upper case representing protein-encoding sequence and the letters in lower case representing untranslated regions. An asterisk (*) represents the stop codon. The underlined letters showed the putative polyadenylation signal (aataaa). The deduced amino acid sequence is shown with blue letters. The sequence was submitted to GenBank with accession number KM514666. (TIF) [file pone.0157591.s001.tif]

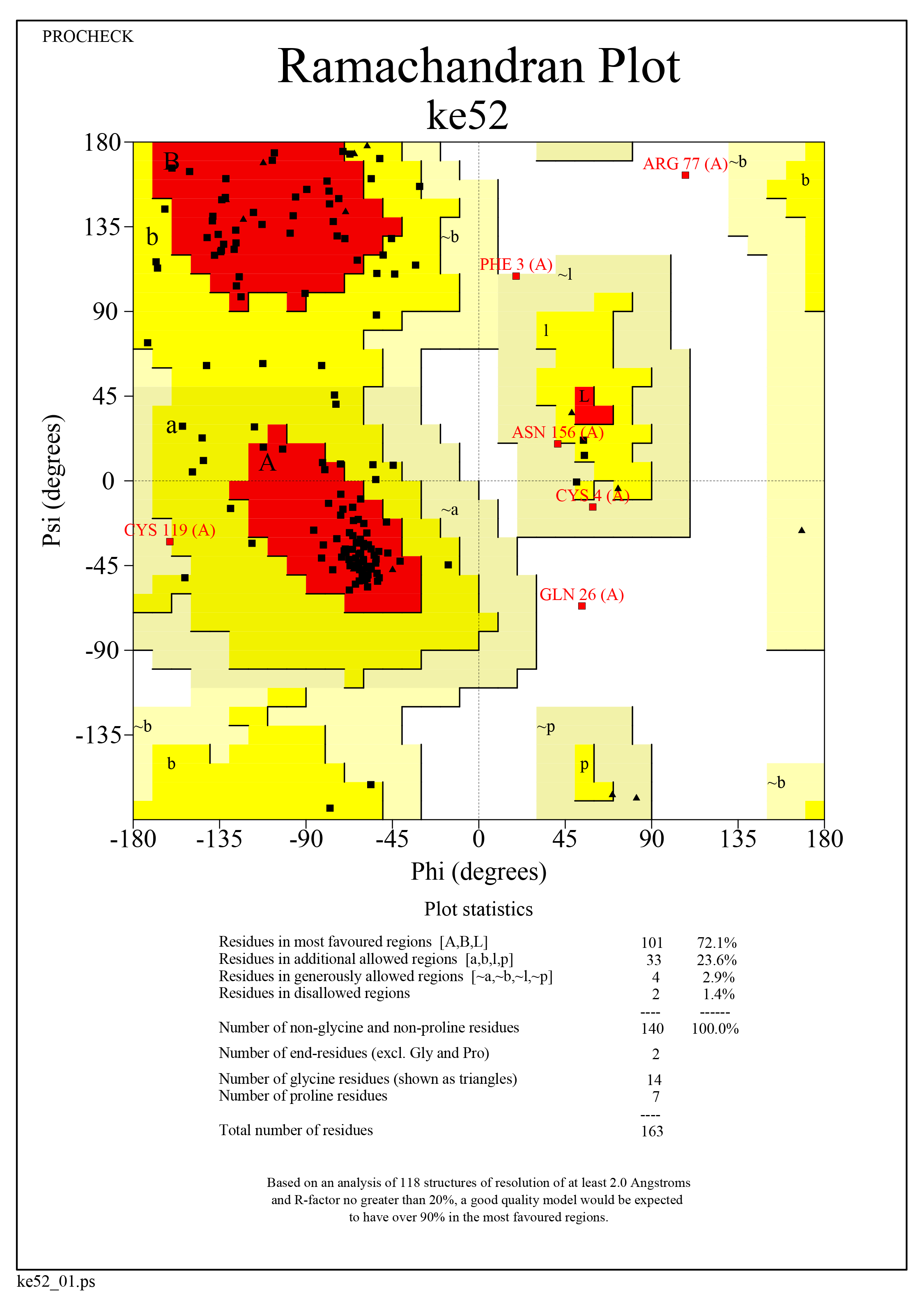

Supplement: S2 Fig — The graphic shows that 72.1% (101 residues) of HbPR-1 amino acids was plotted in the most favored regions, 23.6% (33 residues) in additional allowed regions, 2.9% (4 residues) in generously allowed regions and 1.4% (2 residues) in disallowed regions, respectively. The 85.9% (140 residues) was plotted in non-glycine and non-proline residues, 1.2% (2 residues) represented in the end-residues (excl. Gly and Pro), 8.6% (14 residues) was the glycine residues and 4.3% (7 residues) was the proline residues. (TIF) [file pone.0157591.s002.tif]
